# Retraction: Ribosome-Dependent ATPase Interacts with Conserved Membrane Protein in *Escherichia coli* to Modulate Protein Synthesis and Oxidative Phosphorylation

**DOI:** 10.1371/journal.pone.0326327

**Published:** 2025-06-16

**Authors:** The PLOS One Editors

Following the publication of this article [1], concerns were raised with results presented in Figures 1-4. Specifically,

- When adjusting the color levels to visualize the background, there appear to be repetitive patterns within the backgrounds of the following panels:
  - Figure 1A (i)
  - Figure 2A (i)
  - Figure 4D, upper panel for Spectinomycin (5µg/ml)
  - Figure 4D, upper panel for Gentamycin (2.5 µg/ml)
- In Figure 2A(ii), there are similarities between background regions in the WCL and IP panels.
- The following lanes appear more similar than would be expected from independent results:
  - Figure 1A (ii) IP panel, lanes 1 and 2
  - Figure 2A (ii) IP panel, lanes 1 and 2
- In Figure 3B, the following colonies appear more similar than would be expected from independent results:
  - All colonies shown for *yhjD*Δ::Cm^R^ (donor) *nuoJ*Δ appear similar to those shown for *rbbA*Δ::Cm^R^ (donor) *sdhA*Δ.
  - The bottom right colony of *yhjD*Δ::Cm^R^ (donor) *nuoK*Δ and the bottom left colony of *yhjD*Δ::Cm^R^ (donor) *nuoL*Δ, appear similar to the bottom right colony of *rbbA*Δ::Cm^R^ (donor) *sdhB*Δ and the bottom left colony of *rbbA*Δ::Cm^R^ (donor) *sdhC*Δ, respectively.
  - All colonies shown for *yhjD*Δ::Cm^R^ (donor) *menC*Δ appear similar to those shown for *rbbA*Δ::Cm^R^ (donor) *tolQ*Δ.
- In Figure 3B, the resolution of the *cyoD*Δ colonies in the *rbbA*Δ::Cm^R^ (donor) and *dppC*Δ::Cm^R^ (donor control-III) panels does not appear to match the resolution of other colonies shown in each of these panels.
- In Figure 4D, there appear to be horizontal irregularities suggestive of splice lines in the upper Gentamycin (2.5 µg/ml) panel between the Wild type, *rbbAΔ, yhjDΔ,* and *rbbAΔ yhjDΔ* results.

The first author commented that the appearance of repetitive background elements in the blots may be attributed to technical factors. They provided cropped image data underlying the blots presented in Figure 1 and stated that the original blots underlying the results in Figures 2 and 4 are no longer available. In the absence of high-resolution, uncropped original image data underlying the published panels, the image concerns cannot be resolved.

Regarding the similarity between colonies presented in Figure 3, the first author stated that the colonies appear nearly identical because they were derived from a common parental background and grown on the same non-selective, nutrient-rich medium. The first author commented that the original data underlying Figure 3 are no longer available, and they provided alternative, repeat experiment data for editorial review. PLOS does not consider that repeat experiment data are sufficient to resolve image concerns pertaining to the published panels. In the absence of the original image data used to prepare the published panels, these concerns remain unresolved.

Regarding the horizontal irregularities observed in Figure 4D, the first author explained that the colonies on the plate were misaligned and so minor adjustments were made to align the colony lanes correctly in the published figure.

The *PLOS One* Editors retract this article in light of the unresolved concerns pertaining to the repetitive features in the western blots and the colony plates presented in Figures 1-4, which call into question the reliability and integrity of the published results.

MB, HA, and SP did not agree with the retraction and stand by the article’s findings. WQC, AGagarinova, CG, BL, NS, MJ, AGolshani, AE, and JFG either did not respond directly or could not be reached. MCG is deceased.
